# Developmental Trajectories of Early Life Stress and Trauma: A Narrative Review on Neurobiological Aspects Beyond Stress System Dysregulation

**DOI:** 10.3389/fpsyt.2019.00118

**Published:** 2019-03-11

**Authors:** Agorastos Agorastos, Panagiota Pervanidou, George P. Chrousos, Dewleen G. Baker

**Affiliations:** ^1^II. Department of Psychiatry, Division of Neurosciences, Faculty of Health Sciences, Aristotle University of Thessaloniki, Thessaloniki, Greece; ^2^Unit of Developmental and Behavioral Pediatrics, First Department of Pediatrics, School of Medicine, Aghia Sophia Children's Hospital, National and Kapodistrian University of Athens, Athens, Greece; ^3^Department of Psychiatry, University of California, San Diego, La Jolla, CA, United States; ^4^VA Center of Excellence for Stress and Mental Health, San Diego, La Jolla, CA, United States

**Keywords:** early life stress, trauma, childhood adversity, stress, stress-related-disorders, neurobiology, gene × environment interaction, epigenetics

## Abstract

Early life stressors display a high universal prevalence and constitute a major public health problem. Prolonged psychoneurobiological alterations as sequelae of early life stress (ELS) could represent a developmental risk factor and mediate risk for disease, leading to higher physical and mental morbidity rates in later life. ELS could exert a programming effect on sensitive neuronal brain networks related to the stress response during critical periods of development and thus lead to enduring hyper- or hypo-activation of the stress system and altered glucocorticoid signaling. In addition, alterations in emotional and autonomic reactivity, circadian rhythm disruption, functional and structural changes in the brain, as well as immune and metabolic dysregulation have been lately identified as important risk factors for a chronically impaired homeostatic balance after ELS. Furthermore, human genetic background and epigenetic modifications through stress-related gene expression could interact with these alterations and explain inter-individual variation in vulnerability or resilience to stress. This narrative review presents relevant evidence from mainly human research on the ten most acknowledged neurobiological allostatic pathways exerting enduring adverse effects of ELS even decades later (hypothalamic-pituitary-adrenal axis, autonomic nervous system, immune system and inflammation, oxidative stress, cardiovascular system, gut microbiome, sleep and circadian system, genetics, epigenetics, structural, and functional brain correlates). Although most findings back a causal relation between ELS and psychobiological maladjustment in later life, the precise developmental trajectories and their temporal coincidence has not been elucidated as yet. Future studies should prospectively investigate putative mediators and their temporal sequence, while considering the potentially delayed time-frame for their phenotypical expression. Better screening strategies for ELS are needed for a better individual prevention and treatment.

## Introduction

Stress is defined as the state of threatened homeodynamic balance of the organism (1, 2). Inadequate, excessive or prolonged stress reactions may exceed the organism's natural adaptive capacity and permanently affect stress responses (2, 3). Excessive stress exposure, especially in stress-sensitive developmental stages of higher brain plasticity (e.g., early childhood), may over- or under-sensitize neuroendocrine responses to stress, leading to an altered homeodynamic state (i.e., allostasis/cacostasis) with profound and debilitating effects on physiological development and close association to chronic physical and mental morbidity (2, 4–10).

The term Early Life Stress (ELS) describes a broad spectrum of adverse and stressful experiences (e.g., maltreatment, neglect, separation, parental loss, extreme poverty, starvation, domestic/community/school violence) during the first months of life, early and late childhood and adolescence, while the term has been recently extended by some authors and includes also prenatal life events (11). Childhood Trauma (CT) represents a more specific form of ELS and is defined as “a traumatic event that threatens injury, death, or the physical integrity of self or others and also causes horror, terror, or helplessness at the time it occurs and overwhelms a person's ability to cope” (e.g., physical/sexual abuse, medical trauma, motor vehicle accident, acts of terrorism, war experiences, natural and human-made disasters, witnessed homicides/suicides) (12). ELS/CT constitute a major public health issue, as they occur at ominously high rates, with over 30–40% of the general adult population having experienced some form of disrupting early life adversities (13–16).

In addition, many studies report a negative association of ELS/CT with general adult mental and physical health-related quality of life (17–21). Especially an increased risk for mental disorders (e.g., depression, post-traumatic stress disorder, schizophrenia) and their unfavorable outcomes after ELS/CT experience has been repeatedly reported in several retrospective (5, 22–25) but also prospective studies (26–29). Similarly, history of ELS/CT has been associated with risk behavior patterns, such as substance abuse and suicide attempts in later life (30–35). Furthermore, several larger-scale studies and meta-analyses also suggest a close association of ELS/CT with adverse physical health and in particular with cardiovascular, gastrointestinal, neuromusculoskeletal, pulmonary, inflammatory, and metabolic diseases, chronic pain syndromes, frequency of medical consultations, as well as number of medical diagnoses (24, 36–42).

ELS/CT rarely occurs as a single event but frequently consists of continued maltreatment involving one or more malicious acts. In addition, in most cases, several negative risk factors may co-exist (e.g., poverty, parental absence and parental mental disease, drug addiction) leading to a multifaceted context of multiple chronic stressors. The severity of physical and psychological consequences may be also associated with the number of experienced ELS/CT events (13, 17, 43, 44). More recent studies confirmed that increasing number of ELS/CT may result in higher adult risk for psychopathological complexity and severity, mental comorbidities, prescribed psychotropic medication, poor mental and physical quality of life, as well as several physical conditions (e.g., chronic pain syndromes, cephalgias, heart disease, asthma, diabetes mellitus, and arthritis) (23, 24, 45–50). Apart from number of ELS/CT experiences, the specific nature of ELS/CT and particularly its exact timing could greatly influence downstream biological pathways. Furthermore, genetic factors, presence of caregivers and psychological support, family history of major psychiatric disorders, as well as additional traumatic stress experiences in adulthood may all further influence the individual vulnerability for later disease (51). The continuum of trauma-provoked aftermath reaches from healthy adaptation with high resilience, to severe maladjustment with co-occurring psychiatric and physical pathologies in children, adolescents, and adults. Despite the resilience of many abused individuals in their early years, ELS/CT significantly increases the risk for impaired physical and psychological well-being and adaptive functioning in adulthood.

All these findings suggest that ELS/CT may trigger a health-related risk cascade and be conceptualized as a common developmental risk factor and cumulative health risk mediator, associated with an increased physical and mental morbidity and all-cause mortality in later life (13, 15, 36, 52–59). Although prospective findings support the causal relation between ELS/CT and its long-term adverse health-related effects, so far, little is known about the exact pathways through which ELS/CT is translated into health risk. Observational human studies and experimental animal models suggest that ELS/CT is related to remarkable functional and structural changes even decades later in adulthood. The current hypothetical model suggests that ELS/CT may trigger enduring systemic alterations of fundamental, mainly brain-related plasticity mechanisms and so enhance the biological embedment of distinct “biological memories” of ELS/CT during the sensitive period of early organism development, thus enhancing disease vulnerability in later life (60). To date, most studies assessing the link between ELS/CT and adult disease risk suggest stress system related neuroendocrine alterations as the main pathway of disease development. However, many other related, but distinct biological systems may also play a role and have lately emerged as important pathophysiological pathways.

This current review discusses additionally further potential pathophysiological mechanisms exerting the enduring adverse effects of ELS/CT and mediating the cumulative long-term risk for disease vulnerability in later life, a topic that cannot yet be approached via systematic reviews. Therefore, the literature is presented as a narrative review, providing an overview on the most relevant and acknowledged neurobiological findings from mainly human research. Literature searches were undertaken using PubMed/Medline, PsychINFO, Scopus, and Google Scholar from inception to March 2018 to identify publications (reviews, discussion papers, clinical, observational, and preclinical studies, etc.) addressing neurobiological aspects of ELS/CT and relevant information was extracted. Additionally, the search was complemented through manual review of related terms and citations from article reference lists. The ten most important neurobiological concepts, as backed from current evidence, were synthesized under the headings reported in this narrative review.

## The Human Stress System

ELS/CT can irreversibly disrupt vital central neurobiological systems during vulnerable human development periods and lead to sustainable alterations in stress regulation and psychophysiological reactivity (13, 15, 36, 52–59, 61–63). Because of their pivotal role in the regulation of the dynamic stress response and perhaps also due to a historical focus on these two systems, the hypothalamic-pituitary-adrenal (HPA) axis and the locus ceruleus/autonomic nervous system (LC/ANS) have been more investigated and are considered more crucially affected by ELS/CT than other biological systems (64–66).

### Hypothalamic-Pituitary-Adrenal (HPA) Axis

The chronic dysregulation of the HPA axis is of vital importance in the pathophysiology of stress-related disorders. However, our understanding is hampered by complex and often conflicting relations between HPA axis markers and history of ELS/CT (i.e., findings on both increased and decreased HPA axis activity) (2, 64), as well as the broad definition of ELS/CT (i.e., broad time window of 0–18 y.o.a.). For example, positive ELS/CT history has been repeatedly associated with HPA axis hyperactivity in adults patients with depression and anxiety, but also in healthy individuals [e.g., higher circulating cortisol levels, enhanced cortisol awakening response (CAR), increased adrenocorticotropic hormone (ACTH) and cortisol responses to psychosocial stress or endocrine challenges] (67–75). Chronic hyperactivation of the stress system is related to hypersecretion of corticotropin releasing hormone and arginine-vasopressin (CRH, AVP) by the hypothalamus and ACTH hypersecretion by the pituitary (76), resulting in higher circulating cortisol levels due also to an “insensitive” negative glucocorticoid (GC) feedback of the HPA axis loop (77). The typical example of chronic HPA axis hyper-activation is depression (2, 78), while other conditions such as anorexia nervosa, obsessive-compulsive disorder, panic disorder, alcohol withdrawal, excessive exercising, poorly controlled diabetes mellitus, and hyperthyroidism amongst others, are also associated with increased cortisol levels and HPA axis hyper-activation (79).

On the other hand, several ELS/CT studies have reported HPA axis hypo-activity (e.g., lower circulating cortisol levels, blunted cortisol stress responses) in similar populations and study designs (80–84). This diminished activity could represent a compensatory physiologic adaptation possibly related to a negative feedback hypersensitivity of GC by an up-regulated leukocyte GC-receptor (GR) number and sensitivity (5, 63, 85–87), downregulated secretion of CRH/AVP to the pituitary (76) or a long-lasting GC catabolism drop leading to higher active cortisol persistence in liver and kidney without elevation in the periphery (88). This seems to be the case for patients with post-traumatic stress disorder (PTSD), atypical depression, chronic fatigue syndrome, fibromyalgia, and hypothyroidism.

These results suggest a particularly vital role of GC-signaling in the pathophysiology of ELS/CT (89–91). Insufficient multilevel GC-signaling (resulting from either hyper- or hypo-activation of the HPA axis), may have comparable deleterious effects on the organisms' physiology, as for example seen in the development and long-term effects of both PTSD (i.e., HPA axis hypo-activation) and melancholic depression (i.e., HPA axis hyper-activation) (91–93). These effects appear even greater in individuals with ELS history, suggesting a developmental programming through GC signaling.

Thereby, several factors may have influenced study findings, such as the exact subtype and nature of trauma, sex, the timing and duration of exposure and the assessment of phasic (e.g., diurnal saliva cortisol, cortisol reactivity to challenge) vs. time-integrated cortisol values (e.g., hair cortisol) (94, 95). However, probably the most significant factor modulating the ELS/CT impact on future HPA axis activity may be its exact timing, suggesting a degree of developmental programming through GC signaling.

### Timing of ELS/CT and Developmental Programming of HPA-Axis

The HPA axis activity trajectory develops from infancy to early adulthood and beyond. Together with the HPA axis, however, the amygdala and the hippocampus also develop during the same period following non-linear patterns until early adulthood (96–100). Thereby, periods of greater HPA axis plasticity may represent specific periods of greater vulnerability (96, 100, 101), while mounting evidence suggests a differential impact of ELS/CT on HPA axis activity according to the specific developmental age of exposure (102).

Infancy and early childhood (age 0–5 y.o.a.) represent one of the most vulnerable periods in brain development (6, 101, 103, 104). After an initial hyper-responsive period, the HPA axis may later transition into a stress hypo-responsive period (SHRP) with lower basal cortisol levels and blunted stress-induced cortisol reactivity (101, 104–106). Some longitudinal studies suggest that stress responsivity in early childhood decreases with age throughout the preschool period (101, 105–107), suggesting a potential social buffering of the HPA axis by a nurturing caregiver, who may operate as a safety signal (108–110). This could partly rely on important interactions of GC-signaling with oxytocin pathways, as recently reviewed by Struber et al. (111). Accordingly, this shift from a hyper- to a hypo-responsive stress axis in the first 5 years in life may be represent an particularly crucial stress-sensitive period, especially in the absence of a nurturing caregiver (104). ELS/CT together with higher cortisol during this SHRP could possibly lead to GR insensitivity through greater exposure to GC over time, thus altering the physiological of HPA axis development (101, 112). Studies from Kuhlman et al. (94, 113) confirmed that ELS/CT exposure in the first 2 years in life is associated with prolonged cortisol reactivity to acute social stressors among adolescents.

The later developmental stage of puberty/adolescence represents the second particularly sensitive and vulnerable period with a new major change in HPA axis activity. In this phase, the HPA axis transitions from hypo-responsivity into a period of increased activity (101, 114–116) with progressive higher basal (106, 110, 117, 118) and reactive (106, 118–121) cortisol levels. Interestingly, parental support no longer buffers HPA axis reactivity during this developmental stage (110). On the other hand, it is rather sexual maturation, in interaction with sex and environmental cues, which represents a new major confounder of HPA axis reprogramming (113). The onset of gonadal hormone production plays a vital role in stress and HPA axis reactivity, since estrogen secretion influences GC hyperactivity (122). Some studies on ELS/CT during adolescence reported lower baseline cortisol (123) and blunted cortisol responses to psychosocial stress (124), accordingly suggesting an opposite effect of ELS/CT on HPA axis basal activity and reactivity than in infancy and early childhood.

Summarizing, ELS/CT during the first hypo-sensitive 2 years of life may lead to a hyper-activity and -responsiveness of HPA axis, while ELS/CT during the hyper-active phase of adolescence to a hypo-active and hypo-responsive HPA axis (101). Bosch et al. (125) confirmed this hypothesis showing a relation between ELS/CT in the first year of life, but not late childhood or adolescence, and higher cortisol reactivity in adult life. They could also show higher adult cortisol levels after ELS/CT experience during childhood, but lower cortisol output after experience of ELS/CT in adolescence. These age-dependent differences in HPA axis plasticity could be also reflected on the specific risk for a mental disorder in adulthood. Taken together, ELS/CT exposure in early childhood leads to a similarly higher risk for developing major depressive disorder or PTSD in adulthood, while after ELS/CT exposure in adolescence, the risk for PTSD is greater than for depression (22).

### Locus Ceruleus/Autonomic Nervous System (LC/ANS)

The LC/ANS is also vitally implicated in the stress-related pathophysiological trajectories of trauma (126). LC/ANS and HPA axis are closely interconnected at several neuroendocrine levels throughout the brain and body and their activity normally shows a certain degree of analogy and complementarity. The appropriate regulation of the HPA axis depends at least in part on LC/ANS, especially on vagal influences (127). HPA axis and LC/ANS are both integrated components of an internal neural regulation system (central autonomic network, CAN) (128). Dysregulation of the CAN (129–131) may affect downstream autonomic core centers (i.e., PFC, amygdala, hypothalamus, brain stem nuclei), and alter peripheral ANS activity and overall stress responsivity (130, 132, 133). The significant overlap of the fear/arousal circuitry with the CAN (134) could be, at least partly, responsible for ELS/CT-related autonomic dysregulation. The very high comorbidity of stress- and trauma-related disorders and cardiovascular disease (135–140) confirms a central pathophysiological link between the stress axis and ANS (141–143).

With respect to ELS/CT in particular, a limited number of studies have reported altered autonomic activity in adults with ELS/CT exposure. For example, Otte et al. (144) reported higher catecholamine responses to psychological stress in police recruits, while O'Hare et al. (145) found higher rates of syncope frequency in adulthood in individuals with ELS/CT experience. Heleniak et al. (146) reported blunted cardiac output reactivity and increased vascular resistance associated during a social stress task in ELS/CT-exposed adolescents. However, most studies assessing ANS activity in adult population after trauma included PTSD patients with adult exposure to traumatic stress, repeatedly suggesting an increased sympathetic and/or decreased vagal activity in sequel of a trauma (147).

Some pediatric studies have also lately tried to better investigate the interplay of HPA axis and ANS after ELS/CT. For example, De Bellis et al. (148) reported significantly higher 24 h urinary concentrations of catecholamines in sexually abused girls in comparison to matched controls. Another pediatric study by Gordis et al. (149) reported an asymmetry between the HPA axis and ANS reactivity to a social stressor with absent associations between the peripheral biomarkers of HPA axis (cortisol) and sympathetic activity (salivary alpha-amylase, sAA) only in the maltreated group. In a study longitudinally assessing children after trauma exposure to a motor vehicle accident, Pervanidou et al. (150) could show a successive normalization of cortisol levels but continuously higher catecholamine levels 6 months after trauma exposure, suggesting a lifted cortisol-mediated restraint on catecholamine responses leading to a mid- and long-term enhanced ANS activity. Lower cortisol levels and higher ANS activity found in adult PTSD patients and after ELS/CT exposure may, thus, represent a resulting state of a progredient stress-axes divergence in trauma-related disorders (151). Accordingly, Pervanidou et al. (152) proposed that such a progredient divergence of the two limbs of the stress system following ELS/CT, may represent a vital pathophysiological pathway leading to the long-term impact of ELS/CT on health and the chronic preservation of related symptoms.

## Immune System and Inflammation

Inflammation is a natural immune response to pathogens and injury, an integral part of the stress response and, thus, crucial to tissue healing, adaptation and survival (4, 153, 154). Acute stress activates the secretion of pro-inflammatory cytokines, presumably by adrenergic and CRH-peptidergic stimulation, which help orchestrate the further immune response (e.g., stimulation of systemic acute-phase proteins, such as C-reactive protein, CRP) (4, 155). Pro-inflammatory cytokines, however, unfold systemic effects far beyond the canonical immune response and also stimulate the secretion of CGs, while CGs, in turn, among their numerous pleiotropic effects, help terminate the inflammatory response (153, 154, 156, 157). This is part of a very complex, two-way neuroimmunoendocrine interaction between the central and peripheral limbs of the stress system and the immune axis (156, 158, 159). Growing evidence, accordingly, implicates the immune system in stress resilience and coping through peripheral and central immune mechanisms of action, affecting the brain and all stress-related neurobiological and neuroendocrine responses (160). Vice versa, a dysregulated stress system could allow a disinhibition or excessive inhibition of inflammatory processes, promoting biological aging, inflammatory-related or immunosuppressed medical conditions and compromised overall health (63, 89, 161–163). There is growing evidence suggesting that positive ELS/CT history is an independent risk factor for peripheral immune dysregulation and long-term, low-grade inflammatory excess (i.e., a pro-inflammatory phenotype) in adulthood (101, 164–172).

Given this, the dysfunctional neuroendocrine interface following ELS/CT may be closely correlated to immunological alterations and related long-term health consequences (4, 36, 101, 153, 154, 172, 173), while adults with ELS/CT experience could be at increased risk of disease with potentially immune origin (36, 53). Most human research has been concentrating on pro-inflammatory cytokines and CRP for the immune status characterization. Among all cytokines assessed, interleukin-6 (IL-6) findings are the most robust.

### Interleukin-6 and CRP

IL-6 is a pleiotropic cytokine and simultaneously one of the most suitable inflammatory markers for the characterization of inflammatory status in humans (174), but also an applicable stress biomarker (155), as IL-6 may have a reciprocal modulatory effect on the stress system (175). Indeed, animal and human research confirms that IL-6 stimulates the HPA axis at hypothalamic, pituitary and adrenal level (176–183). Basal IL-6, through activation of the JAK/STAT3 signaling cascade, is required for the sustained cortisol response to chronic stress and is therefore a possible mediator of HPA axis plasticity, in particular in chronic stress states (184). Conversely, cortisol exerts a mild inhibitory effect on the peripheral production of IL-6 (185) and is a major moderator of circadian IL-6 changes (186, 187), while prednisone administration flattens the diurnal rise of IL-6 in the early morning (188). Norepinephrine and epinephrine, on the other hand, lead to an increase of plasma IL-6 in both humans and rats (189–191), in part via beta-adrenergic receptor mechanisms regulating hepatic and splenic IL-6 production (192–194). A recent animal finding also suggested that basal IL-6 signaling in the hypothalamus is a potential determinant of plasticity in the HPA axis response, specifically during chronic stress exposure (184), suggesting that both central and peripheral IL-6 play crucials role on the development of stress susceptibility and related behaviors (175, 195). Several studies have reported dysregulated IL-6 levels in individuals with ELS/CT experience. Carpenter et al. (169) reported higher IL-6 baseline concentrations and a higher inflammatory IL-6 response to acute psychosocial stress challenge in healthy adults with a history of ELS/CT. Using the same paradigm (Trier Social Stress Test; TSST), Pace et al. (196) have shown the same exaggerated IL-6 response to an acute psychosocial stressor in depressed male patients with positive ELS/CT history, compared to depressed patients without ELS/CT history. Interestingly, Kunz-Ebrecht et al. (197) reported an inverse relation between IL-6 and cortisol release to mild mental stress challenges, while Pervanidou et al. (150) provided evidence that IL-6 was involved in the initial biological alterations in the aftermath of trauma, and predictive of PTSD development 6 months later in a longitudinal study design following motor vehicle accidents in children. Finally, in one of the few large (over 3,500 children) prospective studies, Slopen et al. (198) reported ELS/CT being associated with increased levels of IL-6 years later.

With respect to CRP, there are a large number of studies reporting on the association of ELS/CT with increased circulating CRP levels. Most findings, but not all, suggest a robust correlation between ELS/CT and adult CRP levels (165, 166, 170, 199–202). In their seminal study of a birth cohort followed to age 32 years, Danese et al. (165) reported an independent effect of ELS/CT on adult inflammation and suggested that more than 10% of the low-grade inflammation cases in the population may be attributable to ELS/CT. In their prospective study, Slopen et al. (198) found that ELS/CT is a significant independent predictor of persisting inflammation almost 10 years after ELS/CT exposure. Finally, a recent meta-analysis, including over 20,000 samples, confirmed that individuals exposed to ELS/CT show significantly elevated baseline peripheral levels of CRP, IL-6 and TNF-α (203). This study also suggested that specific types of ELS/CT may have differential impacts on single inflammatory markers.

### Neuroimmune Pathways

Although numerous neurobiological links between ELS/CT and inflammation have been put forth, the underlying mechanisms are still not completely understood (159). On the one hand, ELS/CT-related autonomic imbalance with reduced vagal activity may further directly augment inflammation through a direct vagal efferent effect of autonomic brain regions (204–206). On the other hand, HPA axis dysregulation in ELS/CT affects GR-mediated transcriptional and post-transcriptional responses of immune-related genes with lower recovery ability (89, 207). Preclinical research has shown GC resistance in immune cells following repeated acute stress (208, 209), while in humans, prolonged or chronic stress leads also to GR insensitivity of immune cells and, respectively, altered GC inhibitory signal (112, 210). Respectively, several recent human gene expression studies show accumulating evidence for innate immune dysregulation after trauma and a particular and specific (i.e., comorbidity-independent) role of cytokines (211–215). Smid et al. (175) have recently reported both higher mitogen-stimulated T-cell cytokine and innate cytokine production with increasing PTSD symptoms, suggesting a direct effect of cytokine production in stress sensitization. Further human PTSD research suggested that elevated expression of pro-inflammatory cytokines after traumatic stress exposure is probably regulated by multiple epigenetic mechanisms, including dysregulation of microRNA expression (216–218). Interestingly, animal findings suggest that pro-inflammatory cytokines also mediate chronic, stress-induced impairments in hippocampal neurogenesis (167), suggesting that ELS/CT-related subsequent pro-inflammatory diathesis could impair neurogenesis in vital central nervous system (CNS) areas during critical developmental periods and result in a reduced hippocampal volume (see below) and a related malfunction of the fear response circuit in context-dependent situations in adulthood.

## Human Microbiome and the gut-Brain-Axis

During the last decade, the human microbiome and the microbiota-gut-brain (MGB)-axis have become a novel epicenter in mental health and specifically stress-related research and have been already acknowledged as a potentially vital new determinant in the field of neuroimmunoregulation, brain development and behavior (219–223). The MGB-axis represents a bidirectional, key communication pathway between the immune system and the CNS, thus partly mediating the regulation of stress response and early life programming of the neuroimmune system (221, 224). The gut microbiota is a complex ecosystem with a great organism variety and refined genomic structure that resides in the intestinal tract and has a central position in human health and disease (225).

The microbiome produces directly and indirectly significant amounts of antimicrobial peptides, hormones, short chain fatty acids, vitamins, and several neurotransmitters (e.g., 5-HT, catecholamines) and strongly influences our metabolic, endocrine, immune, and CNS (219). In addition, a special role of macrobiota wall constituents on CNS function and development has been suggested recently. For example, peptidoglycans and lipopolysaccharides have been shown to cross the intestinal epithelial barrier and to bind to specific pattern recognition receptors and lead to an activation of the central and peripheral immune system and HPA axis (226, 227). Furthermore, gut microbiota may modulate CNS microglia maturation and functioning and thus also affect neural circuitry of the developing brain (228, 229).

The other way around, the CNS can also modulate the composition and balance of the intestinal microbial community (and mostly Gram-negative bacteria) through the stress system (ANS, HPA axis), (230). For example, PTSD patients show differences in the total abundance of specific bacterial taxa in comparison to trauma-exposed controls (231), while chronic social defeat stress animals models have also lead to shifts in intestinal microbiota composition (232, 233). A chronic bacterial dysbiosis weakens the intestinal mucosal barrier and affects intestinal permeability (“leaky gut”) (234), which possibly results in a microbiota-driven proinflammatory state (235). Thus, a major candidate source of systemic stress-related inflammation could be the disordered gut barrier function (236). A stress-driven microbiome imbalance could then feedback and affect brain functioning by reprogramming the HPA axis through cytokines-related CRH release in the hypothalamus and elsewhere (224, 237–240).

The human microbiome follows a dynamic trajectory development throughout the lifespan and establishes a symbiotic relationship with the organism early in life. Thereby, the development of the intestinal microbiota occurs in parallel with the CNS, having similar critical windows with rapid and profound developmental changes during infancy, childhood, and adolescence (241). Stress-related disruption of the dynamic host-microbe interaction at these critical periods can lead to alterations of the bacterial colonization of the gut in early life and *vice versa* (242, 243). As the microbiome plays an important role in the programming of the HPA axis and stress reactivity (244), ELS/CT may affect the signaling of the MGB axis in a major fashion and alter not only immune, but also CNS and stress system functioning with lifelong emotional and behavioral consequences (i.e., higher risk of neurodevelopmental disorders) (223, 239, 241, 245, 246).

Taken together, the imbalanced human microbiome might be another vital pathway linking ELS/CT with altered neuroimmune reactions and neurodevelopment, as well as long-lasting effects on general health, behavior, emotions, and cognition (247). Risk and resilience to stress- and immune-related disorders may, thus, depend on the diversity and complexity of gastrointestinal microbiota (229), which could play a pivotal role in the etiology of psychiatric illness and make individuals more susceptible to develop psychopathology after ELS/CT (241, 248, 249).

## Oxidative Stress and Cardiovascular System

### Redox State and Antioxidant Defenses

Oxidative stress (OXS), defined as a disequilibrium between oxidant generation and antioxidant defenses (i.e., an altered redox state), has been proposed recently to link ELS/CT to a higher risk of developing psychiatric but also physical morbidity in general (250). Animal findings confirmed that ELS (e.g., maternal separation) has a significant impact on parameters of OXS in mitochondrial function and has shown an association with reactive oxygen species, mitochondrial glutathione, ATP and cytochrome c release in cardiac tissue (251). Furthermore, decreased levels of superoxide dismutase and catalase activity, as well as higher levels of protein carbonylation have been reported in the brain of adult animals exposed to ELS (252). Human research been successfully replicated similar findings. For example, increased OXS markers (i.e., reduced glutathione peroxidase levels, increased protein carbonylation and total reactive antioxidant potential kinetics, etc.) have been reported recently in otherwise healthy ELS/CT-exposed adolescents (253). ELS/CT may so lead to long-term molecular consequences in the basal antioxidant defenses with elevated systemic levels of OXS, stimulating inflammation and driving oxidative damage and accelerated cellular aging in both the CNS and the periphery of the organism (254, 255).

### Telomere Length

Telomeres are DNA-protein complexes located at the ends of linear chromosomes capping and protecting the genome from damage, while inflammation and OXS have been suggested to reduce telomere length. Telomere length is an emerging marker of biological age and OXS, with shorter length being associated with accelerated biological aging, premature cell death and increased morbidity and mortality from age-related diseases (256). Not only has PTSD been associated with shorter telomere length, but also the experience of ELS/CT (257–260). For example, Tyrka et al. (261) investigated healthy adults with absent Axis-I disorders and reported shorter whole-blood telomere length in association with ELS/CT. In a longitudinal study, Shalev et al. (262) showed higher telomere erosion in children 5–10 years old exposed to more than 2 violent events. Chen et al. (263) reported that greater ELS/CT exposure was associated with reduced telomere length and normal telomerase activity in healthy volunteers. A recent study by Mitchell et al. (264) also found a significant association between father loss and children's telomere length, with the death of father showing the greatest effect, and a 90% greater effect in the children with the most reactive alleles of the 5-HTTLPR gene. Finally, two current meta-analytic studies, confirmed the significant association between ELS/CT and accelerated telomere erosion in adulthood (265, 266). ELS/CT could, thus, possibly partly mediate their long-term biological impact also through shorter telomere length, representing another biomarker of increased cacostatic load (51, 256).

## Oxidative Stress and Endothelial Dysfunction

Emerging epidemiologic evidence strongly supports that ELS/CT is an independent albeit silent risk factor of future chronic cardiovascular risk through various systemic and molecular mechanisms (267–272) and that its effect is particularly heightened among women (273). The recent American Heart Association scientific statement offers a comprehensive review of the literature on the influence of ELS/CT on cardiovascular outcomes (274). Besides genetic, metabolic, autonomic, circadian and inflammatory pathways reviewed elsewhere in this article, OXS-related endothelial dysfunction plays a similarly major role in total cardiovascular risk. Animal findings suggest that ELS/CT-related significant endothelial dysfunction is linked to increased superoxide production (275) and reduced endothelial nitrous oxide system buffering capacity with dysfunctional endothelial Angiotensin II-mediated signaling and sensitization to Angiotensin II-induced vasoconstriction (276).

## Metabolism

The stress system is closely interconnected with metabolism. GCs, as the end-effectors of the HPA axis, stimulate appetite (277), alter insulin and leptin secretion and target tissue effects by increasing body weight through the orexigenic and food reward effect of the hypothalamic feeding signal NPY (278, 279) [an effect inhibited by leptin and insulin (280)]. Consequently, in individuals with ELS/CT history, the disrupted biological background described above promotes a tendency toward a dysmetabolic syndrome (281, 282). Accordingly, in the obese population, rates of ELS/CT exposure are reported to be almost twice as high as in the non-obese population (69 vs. 39%) (283). Furthermore, ELS/CT has been repeatedly found to be independently associated with increased overall metabolic risk (284, 285), obesity and increased visceral fat deposition (286–288), decreased HDL, increased LDL levels and lower HDL/LDL ratio (289, 290), higher triglyceride levels (285), an overall prediabetic state (e.g., impaired insulin sensitivity) (291), reduced T3 levels and abnormal metabolism of thyroid hormones (292), enhanced risk for emotional eating as a self-regulatory coping strategy (293) and higher prevalence of metabolic syndrome (290, 294, 295) in later life, while some studies have suggested a dose-dependent relation in these associations (288, 296).

ELS/CT-induced metabolic derangements, such as hyperinsulinemia and altered insulin sensitivity on exposure to a high energy diet later in life, can be a result of altered peripheral gene expression. For example, the interaction between HPA axis activity and liver 11-beta hydroxysteroid dehydrogenase (11β-HSD1) could modulate both tissue and circulating GC availability, with adverse metabolic consequences (297). In addition, genetic interactions with ELS/CT could influence risk for dysmetabolic consequences. HPA axis related FKBP5 polymorphisms, in combination with ELS/CT exposure predict higher insulin and glucose values in midlife (298). Animal findings suggest that ELS/CT is associated with increased food intake, weight gain, increased deposition of abdominal fat, higher plasma triglycerides levels, n-3 PUFA deficiency, etc. (299).

On the other hand, there is also evidence that ELS/CT can exert a programming effect on the adipose tissue and alter the highly sensitive process of adipogenesis (282), leading for example to alterations in adipokine regulation and higher fat accumulations in mice (300). Leptin is an important, circadially secreted adipokine and a vital regulator of energy homeostasis and metabolism, reward processing, brain development and neuroendocrine and immune function (301). Leptin directly interacts with the HPA axis (302), showing an inverse relation to circulating corticotropin and cortisol in healthy men and exerts an anorexigenic effect in conjunction with inhibition of orexigenic pathways via leptin-responsive hypothalamic neurons (303). The adipose tissue–derived protein adiponectin, is another adipokine that may also play a central role in the metabolic dysregulation after ELS/CT. Adiponectin is decreased in obesity (304), whereas hypoadiponectinemia is related to adverse metabolic and cardiovascular outcomes in humans (305). Prospective pediatric studies of physical injury (i.e., burn, MVA) have shown a persistently elevated insulin resistance index up to 3 years (306) and decreased adiponectin levels up to 6 months after physical stress exposure (152).

Taken together, mounting evidence suggests that stress during critical periods of growth and development disrupts the interplay between the stress, circadian and metabolic system and has permanent adverse effects on body size and composition and is often accompanied by associated lifestyle and nutritional risk behaviors (i.e., physical inactivity, emotional eating, disrupted sleep) (282).

## Sleep and Circadian System

The human circadian system (CS) enables the nyctohemeral organization and coordination of many physiological processes and promotes homeostasis and environmental adaptation (307). The HPA axis activity is closely linked to the CS and displays circadian rhythmicity (308–311). Through various pathways, the central circadian system synchronizes hypothalamic neuroendocrine neurons secreting CRH and AVP, modulates adrenal ACTH sensitivity, stimulates GC secretion and defines the peripheral circadian changes in target tissue GC sensitivity (308, 312–314). Circadian acetylation and deacetylation of the GR, modulated by melatonin, allows for these changes in tissue sensitivity (308, 312, 315, 316). In addition, animal studies demonstrated a circadian regulation of peripheral clock gene oscillation in the adrenal gland (317, 318) confirming a nyctohemeral change in its responsiveness to ACTH. Central and peripheral circadian rhythmicity also modulates ANS control through projections to pre-autonomic neurons of the hypothalamus and is essential for the physiologic diurnal fluctuations seen in humans (319–321). Finally, animal and human studies demonstrate responsiveness of cognitive performance to the CS (322, 323). Memory processing, formation and consolidation are directly influenced by the circadian clock and stress (322, 324, 325). Besides light, an important regulator of CS activity is sleep. Sleep acts synergistically and bidirectionally with the central CS, but also independently to reinstate the internal temporal synchrony (326). Specific sleep stages are associated with CLOCK gene expression in the suprachiasmatic nuclei and are tightly ruled by the CS (326–328).

A critical loss of this timed order across several organizational levels of the organism is defined as chronodisruption and promotes a dysharmony of internal biological systems and appropriate biobehavioral adaptations to external stimuli (329) with short- and long-term pathophysiologic and epigenetic impact (330, 331). Chronodisruption may progressively alter the fundamental properties of brain systems regulating neuroendocrine, immune and autonomic function, similar to ELS/CT-related stress axis dysregulation, and may play a central role in the development of stress-related disorders (328).

Direct and indirect human and animal stress research supports the important supraordinate role of CS on stress system and GCs, linking circadian misalignment in ELS/CT-related pathophysiology and potentially resulting in the extensive co-morbidities of ELS/CT through an impaired homeostatic balance. Some animal (332), but—most importantly—numerous human studies including large cohorts, have repeatedly confirmed that ELS/CT is independently associated with enduring adult sleep disruption including global sleep pathology (i.e., insomnia), as well as specific types of sleep problems, such as shortened total sleep time, prolonged sleep onset latency, decreased sleep efficiency, increased number of awakenings, nightmare related distress, sleep apnea and higher nocturnal activity in a probably dose-response manner (333–344).

Sleep deprivation, which is tightly associated with chronodisruption (326–328), has been recurrently related to HPA axis dysregulation findings, such as a flattened cortisol amplitude, decreased CAR and cortisol reactivity, increased but also decreased diurnal cortisol concentrations and increased CRH levels in humans (345–347). Both animal and human studies show that sleep deprivation is associated with increased sympathoadrenal activity and blunted cardiovascular autonomic rhythmicity and responsiveness, thus representing a key cardiovascular risk factor (347–349). Human and animal sleep deprivation studies have reported hypo-responsive medial-frontal cortical regions, hyper-responsive amygdala, and a smaller hippocampal volume (350–352), as shown in adults with ELS/CT history (see above). Sleep disturbances have been associated with altered CLOCK gene expression in humans, which vitally affects neurobiological response to stress (353, 354). Chronodisruption may, thus, sensitize individuals to stress and increase their vulnerability to stress-related disorders (347, 355).

Numerous human and animal studies suggest that acute and chronic physical and/or psychological stress affects the sleep centers of the brain (356–363). Stress, thus, influences sleep physiology and dream patterns and may cause both immediate and long-lasting sleep disruption (364–366), which may, in turn, enhance maladaptive stress regulation (367). For example, REM sleep disruption immediately after trauma exposure has been associated with higher REM-related sympathoadrenal activity, and represents an important predictive factor for the development of trauma-related disorders in humans (368–370). As sleep promotes memory consolidation, in particular for emotional content, sleep deprivation after stress exposure can affect amygdala-cortical connectivity and disrupt this process (371–373).

Such findings suggest that sleep disruption occurring after trauma exposure may represent a core, rather than a secondary pathway that mediates the enduring neurobiological correlates of ELS/CT (364, 368–370, 374, 375) and that chronodisruption may be the common underlying neurobiologic link (370, 374, 376).

## Genetics and Epigenetics

Genome-wide association studies (GWAS) have identified several disease-associated candidate genes, which, however, explain only a minor part of heritability in such complex disorders. In recent few years, the interest has shifted to the central role of the interaction of specific candidate genes with environmental factors, as well as to gene programming through epigenetic regulation (e.g., DNA methylation, histone modification of chromatin, aberrant expression of miRNA) (377, 378). The combination of specific genetic polymorphism profiles and density or activity of functional sites controlling the human stress axis may increase or decrease the risk of psychobiological maladjustment after exposure to ELS/CT. A thorough understanding of the interaction between genes, environment, DNA methylation patterns (methylome) and subsequent gene expression profiles (transcriptome) is integral to our understanding and treatment of stress-related disorders (378).

## Gene × Environment Interactions

Two of the first ground-breaking human studies investigating the interaction between ELS/CT and gene polymorphisms were conducted by Caspi and collaborators. In the first study, abused children with a monoamine oxidase A (*MAOA*) genotype associated with low levels of *MAOA* expression, were more likely to show antisocial-personality disorder and commit violent crimes in adulthood (379). In the second prospective-longitudinal study of a representative birth cohort, functional polymorphisms in the promoter region of the serotonin transporter (5-HTT) gene (5-HTTLPR) was found to moderate the influence of ELS/CT on depression, with the presence of the short allele being associated with more depressive symptoms, diagnosable depression, and suicidality (380). These findings were later confirmed by Karg et al. (381) and are consistent with the assumption that 5-HTTLPR moderates emotional responsivity to stress in interaction with ELS/CT (382).

More recent findings suggest a vital role of genes involved with HPA axis function and GC sensitivity, in conjunction with exposure to child maltreatment or abuse (383). To date, findings mainly implicate two key genes: the GC response element (GRE) and the CRH-releasing hormone receptor 1 (CRHR1) of the FKBP5 gene (383, 384). The co-chaperone FKBP5 regulates steroid receptors such as the GR, resulting in a resistance (reduced sensitivity) against GCs. As first shown by Binder et al. (385), specific single-nucleotide poly-morphisms (SNPs) of the FKBP5 gene interacting with ELS/CT predict the level of adult PTSD symptoms. An allele-specific demethylation in the GREs of FKBP5 may result in a dysregulated expression of GRs (386). Further clinical studies confirm minor alleles of FKBP5 being particularly sensitive and interact with ELS/CT to increase aggressive behavior (387), suicide attempts (388), and depression (389). The CRHR1 acts as a mediator in initiating the stress response, possibly leading to a hypersensitive negative feedback loop of cortisol. Bradley et al. reported in two separate cohorts, independently, that specific CRHR1 polymorphisms interact with ELS/CT to increase the risk of adult depression (390), similar to Heim et al. (391), while Ben-Efraim et al. (392) reported comparable findings with respect to suicide attempts.

Taken together, *gene* × *environment* interactions of gene polymorphisms may affect the acute biological response to ELS/CT and mediate long-term risk of disease to some extent, most probably through their effects on stress responsiveness.

## Epigenetic Regulation

Epigenetic modifications are dynamic—and to some extend reversible—changes, that mediate the interaction between genetic predisposition and environmental factors through regulating functional expression of genes by decreasing, silencing or increasing gene expression (393, 394). The installment of such epigenetic marks by ELS/CT exposure and its genetic moderation by related factors represents a critical factor for vulnerability or resilience to stress-related disorders and may explain inter-individual variation. The interpretation of epigenetic findings is critical due to the complexity of the epigenetic mechanisms and the large number of involved genes.

ELS/CT exposure has been repeatedly related to epigenetic changes and altered gene expression profiles, particularly in the CNS (e.g., hippocampus, amygdala), thus affecting stress responses and memory consolidation (395–398). There is accumulating evidence for gene programming and epigenetic regulation of specific genes in the pathophysiology of PTSD in humans (399–402). Especially, several GC-signaling-related genes (e.g., GCR gene promoter 1F) are sensitive to traumatic-stress-related epigenetic regulation across the lifespan and may represent useful biomarkers related to the development, symptomology and prognosis of PTSD (403, 404). For example, in a recent human brain autopsy material study, history of childhood abuse was associated with changes in DNA methylation related to the neuron-specific GR (NR3C1) promoter in the hippocampus, suggesting distinct effects of ELS/CT on the epigenetic regulation of hippocampal GR expression (405). With respect to the promoter and exon 1F of the human GR gene Nr3c1, Oberlander et al. (406) showed specific epigenetic effects (gene hypermethylation) and elevated cortisol stress reactivity in the offspring due to maternal depression even during late pregnancy. Other animal findings also suggested ELS/CT-related epigenetic changes in the CNS growth and differentiation-related BDNF gene expression (407), while in a genome-wide blood DNA methylation analysis study by Houtepen et al. (408), a locus in the Kit ligand gene (KITLG; cg27512205) was shown to strongly modulate the relation between ELS/CT and cortisol stress reactivity.

Lately, various studies have investigated large-scale methylation patterns with respect to ELS/CT in cross-sectional settings. Bick et al. (409) reported significant differences in methylation in 72 of investigated 173 genes (responsible for HPA and immune system regulation) in children with and without foster care experience. Yang et al. (410) reported significant differences in methylation in 2,868 CpG sites on genes of all 23 chromosomes with respect to presence of ELS/CT, while Essex et al. (411) described similar transgenerational results in more than 150 of 28,000 CpG sites in a prospective study assessing parental stress and its consequences in their offspring. Interestingly, Mehta et al. (412) found that gene expression profiles of PTSD patients with and without ELS/CT are 98% non-overlapping. Moreover, these changes were mostly mediated by DNA methylation changes to a much larger proportion in the childhood abuse group, suggesting that changes in DNA methylation may exert a much greater impact during early life and possibly reflect differences in PTSD pathophysiology, depending on preceding exposure to ELS/CT.

Taken together, enduring changes in the transcriptome may facilitate the response to early developmental challenges and thus play a central role in the long-term (and sometimes transgenerational) biological trajectories of stress-related disease through programming effects for stress reactivity after ELS/CT exposure (104, 413, 414).

## Structural and Functional Imaging Findings

ELS/CT during critical periods of brain development crucially affects the interaction between developing brain regions and neural circuits, exerts epigenetic influences and alters the functions of the HPA axis and GCs; indeed, it has been associated with remarkable structural and functional brain changes even decades later, in adulthood, defining both vulnerability and resilience (383, 415, 416) [for an in-depth review see (417)]. Studies in animals have shown that elevated levels of GCs and catecholamines may lead to alterations in brain development through accelerated loss of neurons (418), delays in myelination (419), or abnormalities in developmentally appropriate synapse pruning (420). ELS/CT-related remodeling of structure, responsiveness and connectivity of specific brain areas and circuits can accordingly alter behavioral, cognitive, emotional, and physiologic responses (51, 421). For example, as cognitive function is heavily dependent on HPA axis and CG activity, childhood adversity associated with HPA axis dysfunction and GC excess or deficiency can result in diminished cognitive functioning and maladaptive emotional behavior (422). Accordingly, in a human resting activity neuroimaging PET study by Insana et al. (423), ELS/CT was associated with altered frontolimbic adult neural activity in the left orbital frontal cortex and left hippocampus, regions involved in executive functioning and emotional autoregulation, socioemotional processes, autonomic function, and sleep/wake regulation. ELS/CT has been also associated with several altered cognitive function findings, such as poor processing speed, defective executive functioning, and memory deficits (e.g., impaired spatial working memory performance, pattern recognition memory) in adulthood, which in turn might pose risks for the development of psychopathology (424–426).

There have been several additional studies assessing structural and functional brain correlates of ELS/CT, but the results have to be explored with caution, given the complexity of brain function, the simplicity of most study paradigms, the age of ELS/CT and assessment, the specific morbid population (i.e., type of psychopathology) and a number of other parameters not taken into account (427, 428). With respect to structural correlates, ELS/CT is associated with disruptive development and reduced volume of corpus callosum, insula, dorsolateral prefrontal cortex (PFC), orbitofrontal cortex (OFC), anterior cingulate gyrus, and caudate, as well as decreased cortical thickness of medial and lateral prefrontal and temporal lobe regions, and reduced overall brain volume in humans (416, 417, 425, 426, 428–434). A study of Teicher et al. (435), utilizing high-resolution T1-weighted MRI scans to assess network connectivity, also reported substantial changes in the cortical network architecture in these areas in young adults with ELS/CT history. Interestingly, the distinct neural plasticity during development can lead to cortical adaptation with very specific regionally altered cortical representation fields (436, 437) and be potentially protecting from the specific sensory processing of different ELS/CT (417). Thus, experience of sexual abuse has been associated with cortical thinning specifically in the genital representation field of the primary somatosensory cortex, while emotional abuse specifically in regions relevant to self-awareness and self-evaluation (438). Such plastic reorganization may be initially protective under abusive conditions, but may underlie later behavioral problems in the same areas (e.g., sexual dysfunction) and be selectively associated with increased vulnerability to internalizing and externalizing psychopathology (434).

The amygdala and the hippocampus are the two brain structures so far mostly reported to be impaired in adult victims of ELS/CT, suggesting most vital effects of ELS/CT on prefrontal-limbic gray matter. The hippocampus is of particular importance because of its role in cognition, but also its rich density of GR, while the amygdala because of its pivotal role in stress responsivity and the extensive related research in mood and anxiety disorders. There are numerous reports and meta-analytic studies confirming the association of ELS/CT with reduced hippocampal volume in adulthood (416, 417, 428, 430, 431, 433, 439). Interestingly, several studies assessing the effects of ELS/CT on hippocampal volume in patients with MDD, suggested that it is rather the history of ELS/CT than depression which is associated with hippocampal atrophy (440–442). However, hippocampal volume seems to be unaffected in children but not in adults with maltreatment-related PTSD, suggesting an initially volumetrically normal hippocampus with subsequent abnormal disrupted development (443). With respect to amygdala, the results from human studies regarding the volumetric effect of ELS/CT are inconclusive, with some studies reporting reduced volume (416, 428, 430, 444), some differential effects according to specific type of ELS/CT (432, 445), and some even greater amygdala volume (in non-human primates) (446). However, findings are conclusive concerning amygdala responsiveness, as ELS/CT has been repeatedly associated with facial threat- or negative-emotion-related amygdala hyper-responsiveness (416, 417, 447, 448). In addition, some studies even suggested that the relation between ELS/CT and risk for adult depression is actually mediated by this preceding amygdala hyperactivity (448, 449).

Finally, imaging studies have investigated the potential influence of genetics (i.e., specific polymorphisms in candidate genes) on the ELS/CT effects described above (417). For example, van Velzen et al. (444) showed that the magnitude of amygdala atrophy in maltreated individuals was significantly associated with the BDNF Val66Met genotype, while Booij et al. (450) demonstrated that greater peripheral serotonin transporter methylation in smaller hippocampal volume in adults with ELS/CT experience. More importantly, there have been a number of studies suggesting a moderating effect of FKBP5 (451–453) and mineralocorticoid receptor genotypes (454) on amygdala volume, reactivity and connectivity of ELS/CT exposed adults, thus implicating HPA axis-related genes in brain development. Genetic susceptibility may, thus, represent a crucial factor leading to related structural and functional trajectories of ELS/CT on brain development (455).

Taken together, altered amygdala-PFC connectivity with reduced top-down regulation of the amygdala by the PFC, reduced contextual input to the amygdala from the hippocampus, and increased connectivity of the amygdala with the LC (leading to increased limbic activity and PFC dysfunction), all suggest that ELS/CT plays a seminal role in functional and structural changes in the brain that may persist along the lifespan (51, 417, 430). Developmental differences in sensitivity to specific forms of childhood maltreatment may lead to different susceptibility of various brain regions and pathways to maltreatment at different ages (417). These results suggest that previously reported structural and functional findings in adolescent or adult psychiatric disease should be re-evaluated addressing ELS/CT as a potential confounder (417).

## Explanatory Models

The developmental origin hypothesis of evolutionary biology suggests that the origins of adult disease are often found among early-life disruptions of physiological developmental processes, ranging from direct causal associations to complex, interacting environmental effects (58, 456–460). The previous sections confirm that ELS/CT during critical phases of perinatal and juvenile brain development is associated with increased cacostatic load and reduced stress adaptability in adulthood, leading to enhanced vulnerability to several chronic diseases. Consequently, various explanatory models have been suggested during the past decades.

According to the cumulative stress model (diathesis-stress model) put forth by McEven et al. (4), when the accumulation of stressors along the life span exceeds a certain threshold, disease development is enhanced in individuals with higher stress exposure. Gluckman et al. (458, 459) suggested a pivotal role of ELS/CT that could prompt developmental (epigenetic) changes underlying predictive adaptive responses leading to a mismatch between the phenotypic outcome of adaptive plasticity and the ability to cope with current stressors increasing risk for disease (match/mismatch hypothesis). In contrast to the cumulative stress model, the mismatch hypothesis explicitly assumes that ELS/CT may also have advantageous effects by representing a possible source of adaptation, potentially even promoting active coping (stress inoculation) to moderate stressors and, thus, resilience. Similarly, the for-better-and-for-worse model suggested by Belsky and Beaver (461) assumes that genetic susceptibility should be contextually interpreted and, according to the specific environment, could be beneficial or not. Nederhof and colleagues have proposed an integrated model based on programming effects of ELS/CT interacting with individual genetic vulnerability (462, 463). Recently, Daskalakis et al. (104) have expanded this model suggesting a three-hit concept for vulnerability and resilience. Accordingly, vulnerability in a given context is enhanced when failure to cope with adversity accumulates. The interaction of the individual genetic background (hit-1) with ELS/CT exposure (hit-2) results in an evolving phenotype with altered stress axis regulation and sensitivity due to early developmental programming, which, in turn, interacts with later-life challenges (hit-3) to result in a higher or lower vulnerability risk according to the type of challenge experienced. This model underlines the extraordinary plasticity of the brain and suggests that “nothing is written in stone” (464).

## Discussion

Coordination of the stress, immune and circadian systems is essential to individual development, adaptation, survival, and well-being (1, 2, 153). ELS/CT, in interaction with genetic factors, disrupts developmental programming of the related neural circuitry and leads to alterations in neuroendocrine, immune, circadian, emotional, and autonomic (re-)activity, with related structural, functional, and epigenetic modifications both in the brain and peripheral tissues. These persistent structural and functional neuropsychobiological changes as sequelae of ELS/CT could mediate risk for chronic disease in adulthood, and lead to cumulative disadvantages and increased adult physical and mental health morbidity (15, 55, 58, 62). Nevertheless, although most studies support a causal relation between ELS/CT and psychobiological maladjustment in later life, the developmental course of such changes and its temporal coincidence has not been elucidated as yet. Thereby, non-linear patterns in neurodevelopment lead to specific periods of greater stress system plasticity, which represent important vulnerability periods (96, 100, 101). Thus, ELS/CT experience is probably associated with a differential impact on stress system activity according to the specific developmental period of exposure (102). ELS/CT exposure during the first hypo-sensitive 2 years of life may lead to a hyper-activity and -responsiveness of HPA axis and accordingly higher risk for developing depression than PTSD, while ELS/CT during the hyper-active phase of adolescence may lead to a hypo-active and hypo-responsive HPA axis and accordingly higher risk for developing PTSD than depression in adulthood (22, 101).

**Figure 1:**
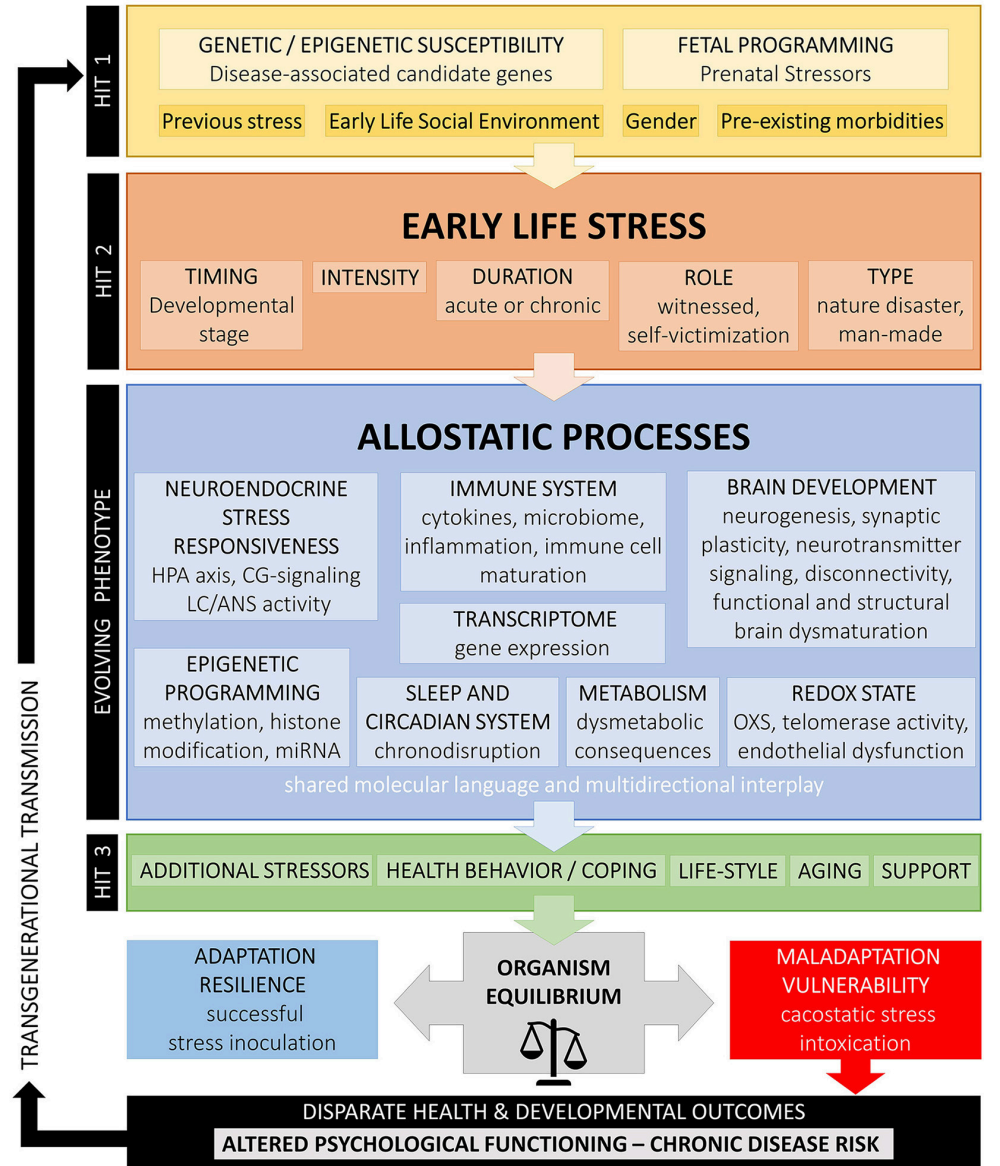
Conceptual model on developmental trajectories of early life stress. Schematic model of moderating factors and allostatic neurobiological trajectory networks involved in the enduring biopsychological effects of ELS/CT. Exposure to ELS/CT can lead to disruption in critical phases of perinatal and juvenile brain development and an evolving programmed phenotype with altered allostatic processes and reduced adaptability to stress. The individual effects on ELS/CT on the organism depend on the specific genetic background and fetal programming (hit-1), the timing, duration, intensity and type of ELS/CT (hit-2) and other later-life challenges, such as additional stressors, coping strategies, support existence, life style, and aging (hit-3). Depending on their interaction, these factors explain inter-individual variation in resilience or vulnerability to altered biopsychological functioning and disparate health outcomes.

Figure 1 summarizes the above developmental approaches and provides an integrative schematic model of moderating factors and allostatic neurobiological trajectory networks involved in the enduring biopsychological effects of ELS. However, further biological pathways (i.e., gonadal steroids, amyloid beta, mitochondrial function, leptin/ghrelin system), psychiatric states (i.e., depression, PTSD), and behavioral patterns (i.e., substance abuse, physical exercise, nutrition) could also play an important role in the mediation of the overall biological risk after ELS/CT and should be better investigated.

## Conclusions

The identification of factors related to risk and resilience in the wake of child abuse is a matter of central importance for public health interventions (465). Understanding the pathways susceptible to disruption following ELS/CT exposure and the effects of a dysregulated interconnection between all neural systems involved could provide new insights into the pathophysiologic trajectories that link toxic stress during developmental stages of childhood and adolescence to adult maladjustment and psychopathology. Future studies should prospectively investigate potential confounders, their temporal sequence and combined effects at the epidemiological, biological, and epigenetic level (466, 467), while considering the potentially delayed time-frame for the expression of their effects. Finally, screening strategies for ELS/CT and trauma need to be improved. Information about ELS/CT history and the number of adverse experiences could help to better identify the individual risk for disease development, predict individual treatment response and design prevention strategies to reduce the negative effects of ELS/CT (468). Detecting and healing of the “hidden wounds” left by ELS/CT should thus be a public health priority.

## Author Contributions

AA managed all literature searches. AA and PP and wrote the first draft of the paper. GC and DB contributed with significant text passages and revised the draft for important intellectual content. All authors have contributed to, read, and approved the final version of the manuscript.

### Conflict of Interest Statement

The authors declare that the research was conducted in the absence of any commercial or financial relationships that could be construed as a potential conflict of interest.
